# Investigation of Blood Characteristics in Nonsyndromic Retinitis Pigmentosa: A Retrospective Study

**DOI:** 10.1155/2019/1902915

**Published:** 2019-05-02

**Authors:** Yijing Yang, Ying Deng, Ye Tian, Zhen Yao, Yasha Zhou, Ying Wang, Qinghua Peng, Jun Peng

**Affiliations:** ^1^Hunan University of Chinese Medicine, Changsha 410208, Hunan Province, China; ^2^Hunan Provincial Key Laboratory for the Prevention and Treatment of Ophthalmology and Otolaryngology Diseases with Traditional Chinese Medicine, Changsha 410208, China; ^3^Department of Ophthalmology, The First Affiliated Hospital of Hunan University of Chinese Medicine, Changsha 410007, Hunan Province, China; ^4^Hunan Provincial Hospital of Chinese Medicine, Zhuzhou 412083, Hunan Province, China

## Abstract

**Purpose:**

To investigate the characteristics of blood in nonsyndromic retinitis pigmentosa (RP) and reveal the pathogenesis of blood cells involved in blood stasis in RP.

**Design:**

This is a retrospective observational study.

**Methods:**

We collected vein blood from 101 cases of patients with nonsyndromic RP and 120 cases of normal individuals according to a single-blind study and used routine clinical examination to detect the indicators of blood. All the subjects were mainly from the central south of China. Data were analyzed statistically between the RP group and normal control.

**Results:**

The indicator of platelet distribution width (PDW) in patients with RP was higher than that in the normal group; the indicators of red blood cell (RBCs), hemoglobin (HGB), hematocrit (HCT), basophils (BASs), platelets (PLTs), and plateletcrit (PCT) in the RP group were lower than those in the normal control. The differences were statistically very significant between the RP group and normal group (*p* < 0.01). There were no statistical differences in the other indicators between the RP and normal group.

**Conclusions:**

The changes in RBCs and PLTs in patients with RP implied that RP induces RBC aggregation and platelet activation, leading to blood stasis which in turn initiates more apoptosis.

## 1. Introduction

Retinitis pigmentosa (RP) is a group of heterogeneous inherited retinal degeneration disease [1]. Extensive studies have been carried out to insight the mechanism in RP [2]. The abnormal blood flows in RP have received considerable attention in recent years, and the findings suggest that attenuation of retinal vessels and decreased blood flow lead to blood stasis in retinal circulation [3–6]. The blood stasis leads to decreased oxygen and material supply which cannot meet consumption for photoreceptor cascade, and thus photoreceptor death occurs [7].

Human blood is composed of plasma and several kinds of cells such as red blood cells (RBCs), white blood cells (WBCs), and platelets (PLTs). Blood performs many important roles within the body, such as supplying oxygen and nutrients to cells and tissues, removing wastes, transporting hormones and signaling, regulating body pH and temperature, and helps in immunological functions and coagulation. Blood rheological behavior affects the vascular system, especially microcirculation and tissue perfusion [8]. Risk factors include increased cell vulnerability and stimulation of local hypoperfusion, hypoxia, and vascular endothelial cells injury, all of which in turn lead to abnormal blood flow [8]. However, the relationship between the characteristics of blood indicators and the pathogenesis of abnormal blood flow in RP is still unclear; understanding the underlying mechanism may help in clinical prevention and treatment of this disease. In this study, we retrospectively analyzed the indicators in clinical routine examination of blood of the nonsyndromic RP patients and normal individuals as control, characterized the blood in nonsyndromic RP, and provided a reference for research of blood flow in RP.

## 2. Methods

### 2.1. Study Design

Our study retrospectively analyzed two datasets of blood samples from patients with nonsyndromic RP diagnosed by First Affiliated Hospital Ophthalmology of Hunan University of Chinese Medicine and Hunan Provincial Hospital of Chinese Medicine from 2012 to 2018. Blood samples were collected from 101 patients with primary nonsyndromic RP and without other system diseases. For these patients, this should be their first medical visit or they have not undergone systemic therapy for RP. The collected 101 samples, including unrelated probands of familial and sporadic cases, were mainly from Hunan, Hubei, Jiangxi, and other provinces, mainly located in the central south of China, including 44 males and 57 females, aged from 8 to 59 (average age of 43.02 ± 13.07). On the other hand, 120 normal individuals who were examined at the physical examination center of the First Affiliated Hospital of Hunan University of Chinese Medicine were selected as the control group, including 63 males and 57 females, aged from 9 to 69 (average age of 40.35 ± 12.50). There was no significant difference in age (*t* = 1.222, *p* > 0.05) and sex ratio (χ^2^ = 1.753, *p* > 0.05) between the two groups. According to the clinical routine blood examination, all blood samples from patients and normal individuals were taken from the vein in the elbow by professionals in hospitals according to the single-blind study.

### 2.2. Diagnostic Criteria of Nonsyndromic RP Patients

A typical case of nonsyndromic RP shows atrophy and pigmentary changes in the retina and RPE, early night blindness, loss of the visual fields, loss of central visual acuity, attenuation of the retinal vasculature, and changes in the optic nerve during the disease [9]. The clinical diagnostic criteria include night blindness, typical “bone spicule” like or irregular pigmentation, retinal vasoconstriction, field of vision reduction such as “tunnel vision,” and abnormal electroretinogram (ERG) or no wave in the whole visual field [10]. RP patients with the following concomitant conditions will be excluded: white spot RP, Stargardt disease, secondary RP, RP with other syndromes, and monocular RP.

### 2.3. Clinical Routine Blood Examination

Two datasets of blood samples from patients with RP were collected by the staffs of both Blood Collection Center from the First Affiliated Hospital Ophthalmology of Hunan University of Chinese Medicine and Hunan Provincial Hospital of Chinese Medicine, and the samples were examined by experienced professionals in their laboratory separately. All patient enrollment information was not informed to the operators, and this is a single-blind study. The blood samples taken were examined using a new automated blood cell analyzer, the Sysmex XE-2100, according to clinical standard testing techniques. We obtained data of routine clinical blood examination including red blood cells (RBCs), hemoglobin (HGB), hematocrit (HCT), mean corpuscular volume (MCV), mean corpuscular hemoglobin (MCH), mean corpuscular hemoglobin concentration (MCHC), red blood cell width variation coefficient (RDW-cv), white blood cells (WBCs), lymphocytes (LYMs), monocytes (MONs), neutrophils (NEUs), eosinophils (EOSs), basophils (BASs), platelets (PLTs), mean platelet volume (MPV), plateletcrit (PCT), and platelet distribution width (PDW).

### 2.4. Statistical Analysis

We used SPSS 22.0 software to perform statistical analysis. Mean and standard deviation were used in two independent samples *T*-tests for comparing RP group with the normal group when the quantitative data were normally distributed; median and interquartile range were used in two independent samples rank sum test for comparing RP group with the normal group when the quantitative data were not normally distributed. *p* < 0.05 was considered statistically significant; *p* < 0.01 was considered statistically very significant.

## 3. Results

### 3.1. Comparison of RBC Indicators between the Normal and RP Group

**Figure 1:**
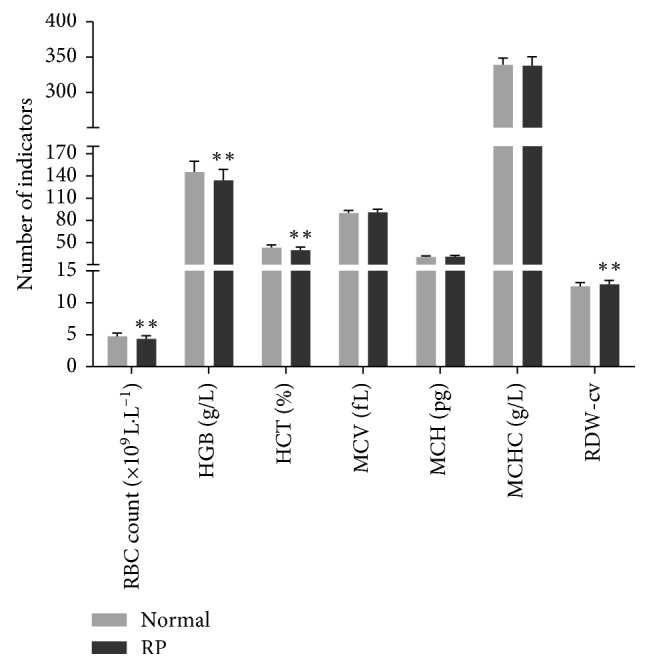
Comparison of RBC indicators between the normal and RP group. All data were in accordance with the normal distribution, and two independent samples *t*-tests were performed for comparison. *p* < 0.05 was considered statistically significant; ^*∗∗*^*p* < 0.01 was considered statistically very significant.

The indicators of RBCs, HGB, HCT, MCV, MCH, MCHC, and RDW-cv both in the RP group and normal control were in accordance with the normal distribution. RDW-cv in patients with RP was higher than that in the normal individuals. The indicators of RBC, HGB, and HCT in the RP group were lower than those of the normal control. The differences were statistically very significant (*p* < 0.01). However, the difference in MCV, MCH, and MCHC was not significant between the RP group and normal group (*p* > 0.05). Data are presented in Table 1 and Figure 1.

### 3.2. Comparison of WBC Indicators between the Normal and RP Group

**Figure 2:**
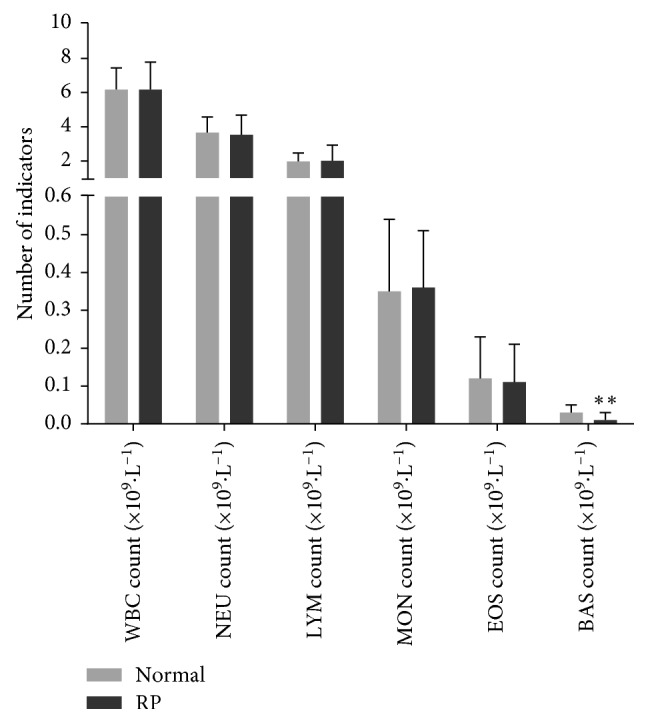
Comparison of WBC indicators between the normal and RP group. Two independent samples *T*-tests were performed for comparing data of WBC and NEU counts. Two independent samples rank sum test was performed for other data. *p* < 0.05 was considered statistically significant. ^*∗∗*^*p* < 0.01 was considered statistically very significant.

The indicators of WBC counts and NEU counts were in accordance with the normal distribution, and two independent *t*-tests were performed for analysis; the other data in the RP group and normal group were both not in accordance with the normal distribution, and two independent samples rank sum tests were performed. BASs in patients with RP were lower than those in the normal control, and the differences were statistically very significant (*p* < 0.01). However, there were no statistically significant differences in the count of WBC, NEU, LYM, MON, and EOS between the RP group and the normal control (*p* > 0.05). Data are presented in Table 2 and Figure 2.

### 3.3. Comparison of PLT Indicators between the Normal and RP Group

**Figure 3:**
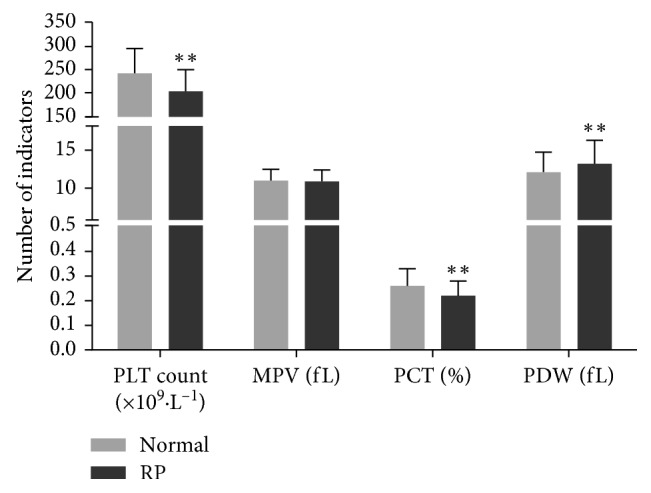
Comparison of PLT indicators between the normal and RP group. Two independent samples *T*-tests were performed for comparing the data of PLT. Two independent samples rank sum test was performed for the other data. *p* < 0.05 was considered statistically significant. ^*∗∗*^*p* < 0.01 was considered statistically very significant.

The count of PLT both in the RP group and normal group was in accordance with the normal distribution, and two independent samples *T*-test was performed. The other data both in RP group and normal group were not in accordance with the normal distribution, and two independent samples rank sum test was performed. PDW in patients with RP was higher than that in the normal control. PLT counts and PCT in patients with RP were lower than those in the normal control. The differences in PLT counts, PCT, and PDW between the RP group and normal control were statistically very significant (*p* < 0.01). There were no statistically significant differences in the MPV between the RP group and normal group (*p* > 0.05). Data are presented in Table 3 and Figure 3.

## 4. Discussion

In this study, we retrospectively analyzed two datasets of blood samples from 101 patients with nonsyndromic RP; these patients included unrelated probands of familial and sporadic cases and were from two hospitals, and they were mainly from the central south of China. One major drawback of this approach is the limited sample size; selection bias is another potential concern because the samples come from the adjacent areas. The indicator of PDW in patients with RP was higher than that in the normal group; the indicators of RBCs, HGB, HCT, BASs, PLTs, and PCT in the RP group were lower than those in the normal group. The differences were statistically very significant (*p* < 0.01). There were no statistically significant differences in the other indicators between the RP group and normal group (*p* > 0.05). The results indicated that RBCs and PLTs may be involved in abnormal blood flow in RP.

Researches commonly suggest that RP sets on with functional or structural abnormality, which further triggers variable pathological mechanisms giving rise to cell death [11]. The mechanisms leading to cell death are expected to disrupt material movement and energy utilization, cause cell vulnerability, stimulate local hypoperfusion, hypoxia, and vascular endothelial cells injury, and further affect blood flow [12, 13]. Retinal blood flows have received considerable attention in recent years due to advances in retinal photographic techniques and image analysis, offering for research on fundamental phenomena and for the development of lab and diagnostic systems [14, 15]. Clinical findings of retinal vessels suggest that microcirculatory changes may be drawn into the progression of RP, especially the loss of central visual function [3]. However, the complex characteristics of blood arising from attenuated vascular and blood stasis remain uncertain, keeping under control the blood stasis, helping in the reduction of peripheral vascular resistance and in increasing blood flow [16].

### 4.1. Indicators of RBCs

The complex characteristics of blood are mainly due to the presence of RBCs which constitute most of the cellular matter of blood [17, 18]. The concentration of RBCs in the plasma, which is the continuous phase of the fluid, plays a part in rendering blood a non-Newtonian fluid. In that case, the thinning and atrophy of retinal and choroidal blood vessels in patients with RP may lead to blood flow in high stress cases, resulting in microcirculatory hypoperfusion, tissue ischemia, and hypoxia [19]. However, the tendency of RBCs to aggregate mainly causes the increase of blood viscosity [16]. RDW-cv is a parameter used in the routine clinical blood examination report to measure the variation of RBC width [20]. Usually, the standard volume of red blood cells is approximately in the range of 6–8 *μ*m. In the case of some variation, of course, the cell volume variable will be significantly affected. The higher the RDW-cv value is, the larger the volume variable will be. In this study, we found indicators of RDW-cv in patients with RP were higher than those in the normal individuals. However, the indicators of RBC counts, HGB, and HCT in RP patients were lower than those in normal controls. RBC aggregation is a physiological phenomenon that takes place in normal blood under low-flow conditions or at stasis. Patients with RP characterized by attenuated vascular results in enhanced aggregation of RBCs and decreased suspension of RBCs in plasma. HGB is the iron-containing oxygen transport metalloprotein in the RBCs. Hemoglobin deficiency can be caused by decreased RBCs and decreased blood oxygen-carrying capacity, both of which are causes of hypoxia [21]. HCT is the volume percentage (vol%) of red blood cells in blood and a point of reference of its capability of delivering oxygen. HCT can indicate possible disease, and an abnormally low HCT may suggest anemia and decrease in the total amount of red blood cells [22]. The increased RDW-cv and decreased RBCs, HGB, and HCT indicate low-flow conditions or stasis and oxygen deficiency in retinal blood flows of patients with RP.

### 4.2. Indicators of WBCs

The number of WBCs in the blood is often an indicator of disease. It is normal when it is part of healthy immune responses which happen frequently and is occasionally abnormal when it is neoplastic or autoimmune in origin. A research has concluded that patients with RP, although not clinically immunologically compromised, have a significantly reduced frequency of T lymphocytes in their peripheral blood due to abnormal expression of HLA-DR antigen on T lymphocytes [23]. The results of comparison of indicators of WBCs in our study also showed that there were no statistically significant differences between the RP group and normal group except BASs, which implies that there was no clinically immunological compromise in patients with RP. BAS cells are chiefly responsible for allergic and antigen response by releasing the chemical histamine causing the dilation of blood vessel [24]. It is appreciated that BASs decreased in the absence of extracellular calcium [25]. In neurodegenerative diseases, calcium ions can make excessive entry into the cell due to the cell toxicity. The excessive calcium in cells may damage the structure or function of cells or even cause cells to undergo apoptosis or death by necrosis [26]. In patients with RP, extracellular calcium ions were fewer for the excessive calcium insults into degenerated photoreceptors or cones and decreased BASs may be detected. In our study, we found that data of BASs in patients with RP were fewer than those of the normal control. However, we need a further study required to shed the light on the mechanism of WBCs affected in RP.

### 4.3. Indicators of Platelets

The main function of the platelets is to contribute to homeostasis. The process of stopping bleeding includes three steps: (i) adhesion: the platelets attach to the interrupted endothelium; (ii) activation: they change shape, turn on receptors, and secrete chemical messengers; and (iii) aggregation: they connect to each other through receptor bridges [27]. When the blood vessels are damaged and contracted, the glycoprotein on the surface of the platelets adheres to the collagen under the endothelium, and the aggregation occurred between the platelets and RBCs. Platelets adhesion to the retina vessel atrophies in RP inciting the platelet consumption and PLT counts decreased [14]. Lower platelet concentration in patients with RP is due to the consumption of platelets. Vasoconstriction also causes a decrease in PCT, which is due to adherence of platelets to the vascular endothelium and accumulation of platelets. There is an increase in both mean platelet volume (MPV) and platelet distribution width (PDW) due to platelet activation. MPV, PDW, and PTC have been investigated as prospective platelet activation markers [28–31]. Mean platelet volume (MPV) is a measurement of the average size of platelets and can be used to make inferences about platelet production in the bone marrow or platelet destruction [28]. PDW is an index used to measure the variation of PLTs' width. MPV and PDW are simple platelet indices, which increase during the platelet activation, and are potentially useful markers for the early diagnosis of blood stasis. PDW is a more specific marker of platelet activation [32]. This study found that PDW in the RP group was higher than that in the normal control; PLT counts and PCT in the RP group were all lower than those in the normal group. The differences in PLT counts and PCT and PDW between the RP group and normal control were statistically very significant. The consumption and activated state of the platelet, which in turn, may further lead to vascular impairment and blood hypercoagulable state in RP patients. The current results suggested that platelets were involved in vascular atrophy and blood stasis in RP.

## 5. Conclusion

The changes occurred in the blood of patients with RP in our research implied that RBCs and PLTs are involved in vascular atrophy and blood stasis in RP. The indicator of PDW in patients with RP was higher than that in the normal control. The indicators of RBC counts, HGB, HCT, BASs, PLTs, and PCT in patients with RP were lower than those in the normal group. In summary, the cell degeneration and abnormal retina vessel in RP induced RBC aggregation, extracellular calcium reduction, platelet consumption, and platelet activation; all the changes lead to blood stasis. It is expected that keeping under control the RBC aggregation and platelet consumption and activation may help in the reduction of peripheral vascular resistance and in increment of blood flow to ameliorate the blood stasis in RP. However, the mechanism of blood stasis in RP remains unknown; the relationship between the characteristics of blood in a hypercoagulable state in RP needs to be further studied.

## Figures and Tables

**Table 1:** Comparison of RBC indicators between the normal and RP group.

Parameters	Normal group (*n*=120)	RP group (*n*=101)	*p* value^*∗*^
RBC (mean (SD)), ×10^9^·L^−1^	4.76 (0.52)	4.38 (0.49)	<0.01^a^
HGB (mean (SD)), g/L	145.28 (14.65)	133.97 (14.94)	<0.01^a^
HCT (mean (SD)), %	42.88 (4.04)	39.51 (4.17)	<0.01^a^
MCV (mean (SD)), fL	89.85 (3.45)	90.67 (4.41)	>0.05^a^
MCH (mean (SD)), pg	30.35 (1.56)	30.72 (1.87)	>0.05^a^
MCHC (mean (SD)), g/L	338.90 (9.64)	337.84 (13.04)	>0.05^a^
RDW-cv (mean (SD))	12.58 (0.57)	12.87 (0.66)	<0.01^a^

^*∗*^ *p* < 0.05 was considered statistically significant; ^*∗∗*^*p* < 0.01 was considered statistically very significant; ^a^*T* test was used to analyze the data.

**Table 2:** Comparison of WBC indicators between the normal and RP group.

Parameters (×10^9^·L^−1^)	Normal group (*n*=120)	RP group (*n*=101)	*p* value^*∗*^
WBC (mean (SD))^—^	6.17 (1.27)	6.17 (1.61)	>0.05^a^
NEU (mean (SD))^—^	3.65 (0.93)	3.54 (1.14)	>0.05^a^
LYM (mean (SD))^—^	1.97 (0.50)	2.01 (0.92)^#^	>0.05^b^
MON (median (interquartile))^—^	0.35 (0.19)	0.36 (0.15)	>0.05^b^
EOS (median (interquartile))^—^	0.12 (0.11)	0.11 (0.10)	>0.05^b^
BAS (median (interquartile))^—^	0.03 (0.02)	0.01 (0.02)	<0.01^b^

^#^Data were expressed as median (interquartile); ^*∗*^*p* < 0.05 was considered statistically significant; ^*∗∗*^*p* < 0.01 was considered statistically very significant; ^a^*T*-test was used to analyze the samples; ^b^rank sum test was used to analyze the samples.

**Table 3:** Comparison of PLT indicators between the normal and RP group.

Parameters	Normal group (*n*=120)	RP group (*n*=101)	*p* value^*∗*^
PLTs (mean (SD)), (×10^9^·L^−1^)	241.97 (53.98)	202.75 (47.06)	<0.01^a^
MPV (median (interquartile)), fL	11.00 (1.50)	10.9 (1.50)	>0.05^b^
PCT (median (interquartile)), %	0.26 (0.07)	0.22 (0.06)	<0.01^b^
PDW (median (interquartile)), fL	12.1 (2.65)	13.20 (3.10)	<0.01^b^

^*∗*^ *p* < 0.05 was considered statistically significant; ^*∗∗*^*p* < 0.01 was considered statistically very significant; ^a^*T* test was used to analyze the data; ^b^rank sum test was used to analyze the samples.

## Data Availability

Dr. Yijing Yang had full access to all the data in the study and took responsibility for the integrity of the data and the accuracy of the data analysis. In order to protect patient privacy, data cannot be made publicly available.
